# Outcomes and Laboratory Predictors of Complications Following Diabetic Foot Amputations: A Ten-Year Tertiary Care-Center Experience

**DOI:** 10.3390/medicina62030525

**Published:** 2026-03-12

**Authors:** Abdulrahman Alaseem, Mishari Alanezi, Othman Alabdullah, Mohamed Ibn Saqyan, Abdulaziz Almanea, Ibrahim Alshaygy, Waleed Albishi

**Affiliations:** 1 Department of Orthopedic Surgery, College of Medicine, King Saud University, Riyadh 12372, Saudi Arabia; 2 College of Medicine, King Saud University, Riyadh 12372, Saudi Arabia

**Keywords:** diabetic foot, amputation, complications, laboratory, albumin

## Abstract

*Background and Objectives*: Diabetic foot disease is a major cause of lower-limb amputation and is associated with substantial morbidity and mortality. While amputation is often considered definitive treatment, postoperative outcomes and their predictors remain incompletely characterized. *Materials and Methods*: This retrospective cohort study included all patients who underwent diabetic foot amputation at a tertiary care center between January 2015 and August 2025. Demographic data, comorbidities, laboratory parameters, and amputation-related variables were collected. The primary outcome was postoperative complications. Secondary outcomes included readmission, re-amputation, ICU admission, length of stay, and mortality. Logistic regression was used to identify predictors of postoperative complications. *Results*: A total of 437 patients were included (mean age 62.0 ± 11.8 years; 65.7% male). Postoperative complications occurred in 52.1% of patients, most commonly infections (31.6%) and acute kidney injury (18.1%). Readmission occurred in 50.6%, re-amputation in 23.4%, ICU admission in 28.7%, and mortality in 17.8%. On multivariable analysis, preoperative serum albumin was the only independent predictor of postoperative complications (aOR 0.944, 95% CI: 0.913–0.976; *p* < 0.001). *Conclusions*: Postoperative complications following diabetic foot amputation are common and associated with significant healthcare utilization and mortality. Preoperative hypoalbuminemia was the only independent predictor of postoperative complications, supporting its utility as an integrated biomarker of systemic illness (including inflammatory burden/infection and hepatic synthetic function) for perioperative risk stratification.

## 1. Introduction

Diabetes mellitus (DM) represents a major global health challenge, with an estimated 537 million affected individuals worldwide in 2021, a figure projected to rise to 783 million by 2045 [1]. With this rising burden, diabetes is increasingly associated with long-term complications, among which diabetic foot disease is one of the most serious. This condition not only reduces patients’ quality of life but also leads to prolonged hospitalizations, long-term disability, and increased mortality [2].

Foot problems in patients with diabetes are common and costly. Studies suggest that up to one in four patients with diabetes may develop diabetic foot ulcers (DFUs) during their lifetime [3]. It is also estimated that 20–40% of all healthcare spending for diabetes is related to managing foot problems [4]. The worldwide prevalence of DFUs is about 6.3%, with men and people with type 2 diabetes being at higher risk [5]. The outlook for patients with DFUs is often poor; around 5% die within the first year of diagnosis, and almost half die within five years [6]. Compared with individuals without diabetes, those with the disease have an approximately 23-fold greater risk of undergoing a lower-limb amputation [7].

The burden of diabetic amputations is increasing, with particularly high rates reported in the Middle East due to the widespread prevalence of diabetes and its associated complications [8]. Saudi Arabia has the second-highest rate in the region and ranks seventh globally, with an estimated 7 million individuals affected [9]. Despite this high burden, most studies have focused narrowly on amputation as the endpoint, with little attention paid to the broader spectrum of postoperative outcomes. Events such as post-operative complications, re-amputations, and mortality at varying intervals remain underexplored. This lack of comprehensive outcome data limits the development of effective risk stratification and targeted preventive strategies for high-risk patients. Therefore, this study aims to address this gap by presenting a decade-long, retrospective analysis of patients undergoing diabetic foot amputation. Specifically, it evaluates the incidence of postoperative complications across multiple time points and investigates demographic, clinical, and laboratory predictors associated with these events.

## 2. Materials and Methods

### 2.1. Study Design and Setting

This retrospective cohort study was conducted at King Saud University Medical City, a tertiary-care center in Riyadh, Saudi Arabia. The institution is a high-volume center that receives referrals from across the country for managing complex diabetic foot disease. We reviewed all consecutive cases of upper- and lower-limb amputations performed between January 2015 and August 2025. Eligible participants included adult patients (aged ≥18 years) of both genders who underwent amputation due to diabetic complications. This includes patients with a documented diagnosis of DM who required amputation for diabetic foot ulcers, gangrene, or infection, where diabetes was identified as the primary underlying cause. Patients who underwent amputation for other causes, such as trauma, malignancy, or congenital abnormalities, as well as those with incomplete medical records, were excluded from the study. The study was approved by the Institutional Review Board (IRB) of the College of Medicine, King Saud University (Project No. E-22-3920). Given the retrospective nature of the study, the IRB waived the requirement for informed consent.

### 2.2. Data Collection and Variables

Data were collected from the institution’s Electronic Medical Records (EMRs) and recorded in a computerized datasheet. Baseline characteristics were collected at the time of the index amputation and included demographic variables (age, gender, marital status, and nationality), as well as anthropometric measurements (weight, height, and body mass index [BMI, calculated as kg/m^2^]). Clinical comorbidities were also recorded, including hypertension, dyslipidemia, coronary artery disease (CAD), chronic kidney disease (CKD), and other relevant systemic conditions. Preoperative laboratory parameters were defined as the closest available results within a 30-day window preceding the index amputation. These included glycated hemoglobin (HbA1c, %), hemoglobin (g/L), serum creatinine (µmol/L), serum albumin (g/L), total white blood cell count (WBC, ×10^9^/L), lymphocyte count (×10^9^/L), neutrophil count (×10^9^/L), and erythrocyte sedimentation rate (ESR, mm/h). Additionally, amputation-related details such as level of amputation, admission type (emergency vs. elective), and surgical specialty performing the procedure were also collected. For analytic purposes, amputations were additionally categorized as minor (toe, metatarsal, and other partial-foot amputations) or major (below-knee amputation or above-knee amputation).

### 2.3. Outcome Measures

Outcomes were assessed from the date of index amputation to either the last documented follow-up or the date of death. The primary outcome was the occurrence of postoperative complications, defined as any adverse event during the index hospitalization or during subsequent readmissions. These included infectious complications (e.g., surgical site infections [SSIs], osteomyelitis), acute kidney injury (AKI), cardiovascular events (including myocardial infarction (MI), stroke, and venous thromboembolism (deep vein thrombosis [DVT] or pulmonary embolism [PE]), and other complications such as bleeding, delirium, electrolyte disturbances, and septic shock. Complication categories were pre-specified prior to data abstraction. Postoperative complications were ascertained through structured review of the EMRs for the index admission and any subsequent readmissions (including operative notes, daily progress notes, laboratory results, microbiology and radiology reports, and discharge summaries) and recorded in the study database. Operational definitions and data sources are summarized in Supplementary Table S1. Secondary outcomes included unplanned hospital readmission, ICU admission, re-amputation, and all-cause mortality. Readmission was defined as any unplanned all-cause admission after discharge from the index amputation and was stratified by timing (30-day, 90-day, and >90-day); where documentation permitted, readmissions were further categorized as amputation-related when primarily attributable to the index procedure or its complications (e.g., stump/wound complications, infection, or reoperation), otherwise as non–amputation-related. Re-amputation was defined as any subsequent lower-extremity amputation after the index procedure (planned staged amputations could not be reliably distinguished from unplanned reoperations).

### 2.4. Statistical Analysis

Descriptive statistics were used to summarize the cohort’s characteristics. Continuous variables were presented as mean and standard deviation (SD) for normally distributed data or median and interquartile range (IQR) for non-normally distributed data. Normality was assessed using the Shapiro–Wilk test and visual inspection of histograms. Categorical variables were presented as frequencies and percentages (*n*, %). For comparative analyses, the chi-square test or Fisher’s exact test was used for categorical variables. The independent samples *t*-test or Mann–Whitney U test was used for continuous variables between two groups (e.g., complication vs. no complication). The distribution of patients’ demographics across the entire population was assessed using a chi-square goodness-of-fit test to evaluate deviation from a uniform distribution. To account for heterogeneity by anatomic level, key outcomes were compared between minor and major amputation categories. To identify independent predictors of postoperative complications, binary logistic regression analysis was performed. Results were reported as odds ratios (OR) and adjusted odds ratios (aOR) with their corresponding 95% confidence intervals (CI). A two-tailed *p*-value of <0.05 was considered statistically significant for all analyses. All statistical analyses were conducted using IBM SPSS Statistics version 26.0 (IBM Corporation, Armonk, NY, USA).

## 3. Results

### 3.1. Demographic and Clinical Characteristics

A total of 437 patients with a mean age of 62.0 ± 11.8 years. The majority were male (65.6%), and nearly half of the patients were aged 60–79 years (50.3%), followed by those aged 40–59 years (38.4%), while only 8.2% were ≥80 years and 2.9% were 18–39 years (*p* < 0.001). The median body mass index (BMI) of the cohort was 28.14 kg/m^2^ (IQR 24.41–32.40). Hypertension was the most common comorbidity, affecting 78.5% of patients, followed by dyslipidemia (43.01%), CAD (42.26%), and CKD (28.41%) (Table 1).

### 3.2. Laboratory and Amputation-Related Details

Laboratory findings revealed a median HbA1c of 9.20% (IQR 7.70–10.88), indicating poor glycemic control across the cohort. The median hemoglobin level was 103.00 g/L (IQR 92.00–116.00), while serum creatinine and albumin levels were 98.00 µmol/L (IQR 70.00–185.50) and 24.75 g/L (IQR 19.76–30.21), respectively. Inflammatory markers were notably elevated, with a median WBC count of 12.70 × 10^9^/L (IQR 8.72–17.80), neutrophil count of 9.50 × 10^9^/L (IQR 5.60–14.60), and ESR of 87.50 mm/h (IQR 54.25–109.00). In contrast, lymphocyte counts remained largely within normal limits, with a median of 1.70 × 10^9^/L (IQR 1.20–2.40). Regarding the level of amputation, the majority of patients underwent toe amputation (45.8%), followed by below-knee amputation (31.8%) and above-knee amputation (17.4%), while metatarsal (3.6%) and other amputations, such as Chopart and Lisfranc, were less common (1.3%). When grouped by amputation category, 50.8% procedures were classified as minor and 49.2% as major amputations. Most procedures were performed in an emergency setting (79.7%). Vascular surgeons performed the majority of amputations (54.9%), followed by orthopedic surgeons (27.6%) (Table 2).

### 3.3. Postoperative Complications

More than half of the cohort developed at least one postoperative complication (52.1%). Infections, including osteomyelitis, stump infections, wound infections, and surgical site infections, were the most common (31.6%), followed by acute kidney injury (18.1%). Other complications included electrolyte disturbances (3.7%), delirium (3.4%), septic shock (3.0%), and stroke (3.0%).

### 3.4. Readmission, Re-Amputation, and Mortality Rate

Among the 437 patients included in the study, the overall readmission rate was 50.6% (*n* = 221), with a calculated cumulative incidence of 0.506 (95% CI: 0.459–0.553). Stratified by timing, 30-day readmissions occurred in 7.7% of patients [0.077 (95% CI: 0.052–0.102)], 90-day readmissions in 12.0% [0.120 (95% CI: 0.089–0.151)], and late readmissions beyond 90 days in 36.9% [0.369 (95% CI: 0.323–0.415)]. Among the 221 readmitted patients, 92 (41.6%) were adjudicated as amputation-related readmissions, whereas 129 (58.4%) were non–amputation-related. ICU admission was required in 124 patients (28.7%) with a cumulative incidence of 0.287 (95% CI: 0.244–0.330), while re-amputation was performed in 23.4% of patients [*n* = 101; 0.234 (95% CI: 0.194–0.275)]. Mortality was observed in 77 patients (17.8%), corresponding to a cumulative incidence of 0.178 (95% CI: 0.142–0.215). The median length of hospital stay was 21.17 days (IQR 10.25–38.63). Outcome rates stratified by amputation category are summarized in Supplementary Table S2. Compared with minor amputations, major amputations were associated with higher rates of ICU admission (39.5% vs. 17.6%; *p* < 0.001) and mortality (24.2% vs. 11.3%; *p* = 0.002). On the other hand, there was no significant difference between minor and major amputations in terms of readmissions (54.9% vs. 46.1%; *p* = 0.063) and re-amputation (27.0% vs. 19.4%; *p* = 0.136).

### 3.5. Characteristics of Patients with and Without Postoperative Complications

**Figure 1:**
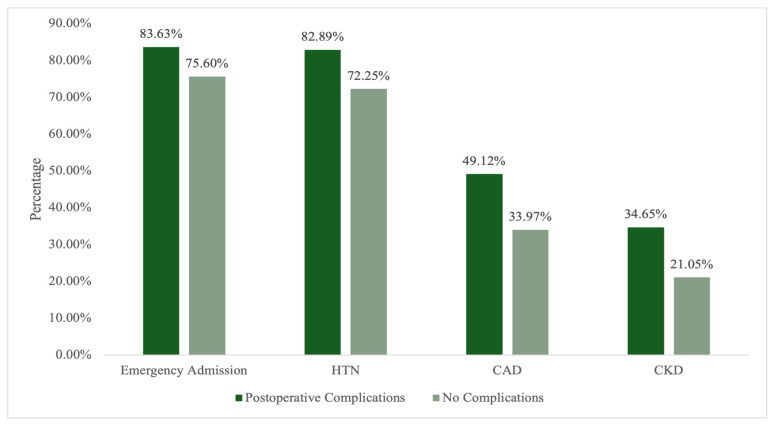
Baseline clinical characteristics in patients with and without postoperative complications; HTN, hypertension; CAD, coronary artery disease; CKD, chronic kidney disease.

There were no significant differences between patients with and without postoperative complications with respect to age, sex, anthropometric measurements, or level of amputation. However, emergency admissions were significantly higher among patients who developed complications (83.63% vs. 75.60%, *p* = 0.037). Regarding comorbidities, hypertension (82.89% vs. 72.25%; *p* = 0.007), CAD (49.12% vs. 33.97%; *p* < 0.001), and CKD (34.65% vs. 21.05%; *p* < 0.001) were all significantly more prevalent in the complication group. Laboratory findings also demonstrated clinically relevant differences. Patients who developed complications had significantly lower hemoglobin (102.0 vs. 105.0 g/L; *p* = 0.005) and albumin levels (22.9 vs. 27.28 g/L; *p* < 0.001), as well as higher creatinine (118.5 vs. 85.0 µmol/L; *p* < 0.001), WBC count (13.6 vs. 11.6 ×10^9^/L; *p* < 0.001), and neutrophil count (10.75 vs. 7.87 ×10^9^/L; *p* < 0.001). Lymphocyte counts were significantly lower among patients with complications (*p* = 0.015), whereas ESR and HbA1c values were comparable between groups. Furthermore, patients who experienced postoperative complications had significantly longer hospital stay (median 26.09 vs. 15.38 days; *p* < 0.001), as detailed in Table 3 and Figure 1.

### 3.6. Predictors of Postoperative Complications

In the univariate analysis, several factors were significantly associated with higher odds of postoperative complications. These included emergency admission (OR 1.655, 95% CI 1.025–2.674; *p* = 0.040) and the presence of hypertension (OR 1.745; *p* = 0.019), CAD (*p* = 0.003), and CKD (*p* = 0.003). Additionally, laboratory parameters associated with increased risk included lower hemoglobin (*p* = 0.001) and lower albumin levels (*p* < 0.001), as well as higher creatinine (*p* = 0.004), WBC count (*p* < 0.001), and neutrophil count (*p* < 0.001). In the multivariate analysis, albumin remained the only independent predictor, with higher albumin levels demonstrating a protective effect against postoperative complications (aOR 0.944, 95% CI 0.913–0.976; *p* < 0.001). All other variables lost statistical significance after adjustment, suggesting that their univariate associations were likely influenced by confounding factors. Additionally, in multivariable analysis, amputation category (major vs. minor) was not independently associated with postoperative complications (aOR 1.20, 95% CI: 0.77–1.86; *p* = 0.425), as shown in Supplementary Table S3.

## 4. Discussion

In this ten-year retrospective study of patients undergoing diabetic foot amputations, postoperative complications were found to be notably common, highlighting the particular vulnerability of this population. This elevated risk reflects the systemic nature of diabetic foot disease, which is often accompanied by chronic inflammation, multiple comorbidities, and impaired wound-healing capacity. Infectious complications were the most frequent postoperative issue, consistent with the existing literature that identifies infection as a major contributor to adverse surgical outcomes in this group. Our findings also highlighted the influence of underlying clinical factors, particularly poor nutritional status and systemic illness, on postoperative outcomes, emphasizing the importance of comprehensive preoperative assessment and optimization.

In our cohort, men constituted nearly two-thirds of the population (65.7%), reflecting the well-documented male predominance in diabetic foot disease and lower-limb amputation worldwide [10]. This pattern has been attributed to a higher prevalence of peripheral neuropathy, greater occupational exposure to foot trauma, and lower rates of health-seeking behavior among men [11,12]. Our findings align with those of Fan et al. [10], who demonstrated in a large systematic review and meta-analysis of more than 33 million diabetic patients that male sex is significantly associated with a higher risk of lower-limb amputation. However, despite this clear disparity in amputation risk, our analysis showed that gender was not significantly associated with postoperative complications, indicating that male sex, while more common among patients requiring diabetic foot amputation, does not itself confer additional postoperative risk. Age distribution in our cohort showed that the majority of patients were between 60 and 79 years (50.3%), aligning with the typical demographic affected by advanced diabetic foot disease [13]. Although advancing age is frequently reported as a risk factor for poor surgical outcomes due to frailty, impaired immunity, and multiple comorbidities, our findings did not demonstrate a significant association between age and postoperative complications. This may reflect the overwhelming influence of other clinical factors, such as comorbidities, nutritional depletion, and systemic inflammation, which appear to outweigh chronological age in determining postoperative vulnerability. The absence of an age effect in our analysis suggests that functional and physiological status may be more relevant targets for risk stratification than age alone in patients undergoing diabetic foot amputation. The high postoperative complication rate observed in our cohort (52%) indicates that amputation for diabetes-related foot disease does not return patients to a lower-risk baseline. Similarly, Bischoff et al. [14] also reported a complication rate of 62.5% following below-knee amputation. Together, these findings suggest that amputation is followed by a postoperative course characterized by persistent systemic vulnerability and markedly limited physiological reserve. Our finding that infections were the most common postoperative complication (31%) aligns with numerous previous studies. For example, Hamudu et al. [15] reported a comparable rate of postoperative infection of 30% among patients undergoing lower-limb amputation. This predominance of infection is biologically coherent with the pathophysiology of the diabetic foot: long-standing peripheral neuropathy and peripheral artery disease (PAD), combined with impaired innate and adaptive immune responses, create a fertile ground for infection that persists even after amputation of necrotic or overtly infected tissue [16,17]. Furthermore, the majority of amputations were performed as emergency procedures (79.8%), with only a small proportion carried out electively. This substantial disparity reflects the acute clinical deterioration often seen in patients presenting with diabetic foot complications. The predominance of emergency interventions suggests late presentation, delayed referral, or rapid progression of underlying infection or ischemia, factors frequently reported in the diabetic limb-threatening spectrum [18]. On univariate analysis, patients who developed postoperative complications in our cohort were more likely to be admitted emergently, to have a heavier burden of comorbidities (notably hypertension, CAD, and CKD), and to present with an adverse laboratory profile characterized by lower hemoglobin and serum albumin, and higher creatinine and WBC count. This pattern reflects chronic anemia, malnutrition, renal dysfunction, and systemic inflammation, a combination widely recognized as a marker of limited physiological reserve and high surgical risk. A similar pattern has been reported in earlier studies. For example, Almohammadi et al. [19] found that low hemoglobin and elevated creatinine were independently associated with mortality among diabetic foot patients, most of whom underwent amputation. Additionally, Shatnawi et al. [20] also showed that poor glycemic control and cardiovascular comorbidities were major predictors of major amputations in patients with diabetic foot, which highlights the central role of macrovascular disease and systemic illness in determining postoperative outcomes. After multivariable adjustment, serum albumin emerged as the only statistically significant independent predictor of postoperative complications, with each 1 g/L increase associated with a 5.6% reduction in the odds of experiencing a complication, supporting a graded association. Although albumin is often used as a marker of nutritional reserve, it is also a negative acute-phase reactant and may be reduced in the context of systemic inflammation and infection, hepatic dysfunction, and fluid shifts; therefore, our findings should be interpreted primarily as identifying hypoalbuminemia as an integrated prognostic marker of overall physiological stress and disease burden, rather than implying a direct causal nutritional effect. In a large cohort of patients undergoing lower extremity amputation (LEA), Chahrour et al. [21]. demonstrated a dose–response relationship between preoperative hypoalbuminemia and postoperative mortality, particularly after major LEA, highlighting low albumin as a powerful prognostic factor in this population. The re-amputation rate of 23.4% observed in our study is a point of concern. This finding aligns with Wong et al. [22], who reported that 38% of patients undergoing below-knee amputation required an unplanned reoperation within one year. This high rate suggests that, in many cases, the initial procedure did not fully eradicate infection, restore adequate perfusion, or secure a reliably healable stump; however, in this retrospective dataset we could not reliably distinguish planned staged amputations from unplanned reoperations, and the observed rate likely reflects a spectrum of persistent infection/ischemia, stump failure, and progressive diabetic limb pathology. Therefore, there is a critical need for meticulous preoperative planning, optimization of vascular and infectious status, and structured long-term multidisciplinary follow-up to minimize the risk of further limb loss. Our all-cause readmission rate of 50.6% reflects the extent to which amputation initiates a cycle of recurrent acute care rather than closing the treatment trajectory, as well as the difficulties of maintaining wound care and glycemic control. Notably, 41.6% of these readmission events were amputation-related, indicating that a substantial proportion of rehospitalizations were driven by postoperative stump/wound complications, infection, or the need for further operative management. This burden is broadly consistent with large registry data after major LEA studies. In a Danish nationwide study, 23–27% of patients were readmitted within 30 days and 35–40% within 90 days [23]. Similarly, a Canadian study by Kayssi et al. [24] reported a one-year readmission rate of 55.4%. These data position our readmission rate consistently across health systems; therefore, a structured post-discharge surveillance and accessible multidisciplinary follow-up pathways are essential. Mortality in our series was also substantial, with an overall death rate of 17.8%. This aligns with the wide mortality spectrum reported following diabetes-related major LEA. A recent population-based study from England involving 28,045 diabetic patients undergoing below-knee amputation demonstrated 30-day, 90-day, one-year, and five-year mortality rates of 7.1%, 12.7%, 24.6%, and 61.2%, respectively [25]. Collectively, these findings emphasize that patients undergoing LEA constitute an ultra-high-risk cohort, warranting intensified long-term follow-up, structured multidisciplinary surveillance, and proactive strategies to prevent additional limb loss and premature mortality. Although the present study focuses on perioperative outcomes after diabetic foot amputation, these findings also highlight the importance of upstream prevention to reduce progression to late-stage limb-threatening disease. Core preventive strategies include structured foot surveillance, patient education, optimization of glycemic control and vascular risk factors, timely referral to multidisciplinary diabetic foot services, and earlier detection of diabetic peripheral neuropathy (DPN) [26]. Several well-established techniques are available for early detection of DPN in clinical practice. Corneal confocal microscopy enables noninvasive visualization of small nerve fiber changes and has demonstrated diagnostic utility in detecting early neuropathic changes before overt clinical manifestations [27]. Skin biopsy with quantification of intraepidermal nerve fiber density remains a validated method for assessing small fiber neuropathy and provides an objective structural evidence of small fiber involvement [28]. In addition, careful assessment of clinical signs and symptoms, including vibration perception, monofilament testing, temperature discrimination, and neuropathic symptom scoring systems, remains an important part of early DPN diagnosis in routine practice [29]. Emerging neuroimaging techniques, such as diffusion tensor imaging (DTI), have shown promise in detecting microstructural alterations in peripheral nerves; however, this technique remains primarily investigational and is not yet validated for routine clinical detection of DPN [30]. Further prospective studies are required before such modalities can be integrated into standard screening pathways.

This study has several important strengths. First, it represents a large, decade-long cohort of patients undergoing diabetic foot amputations at a high-volume tertiary care center, providing a robust sample size and enhancing the reliability of the observed outcomes. Second, the study incorporated a broad range of variables, including demographic characteristics, comorbid conditions, laboratory parameters, and amputation-related details, enabling a detailed exploration of potential predictors of postoperative complications. Additionally, the use of multivariable regression analysis strengthened the identification of independent predictors while accounting for confounding factors. Despite these strengths, several limitations should be acknowledged. First, the retrospective design inherently limits causal inference and is subject to selection bias and information bias, including reliance on the accuracy and completeness of EMRs. Although patients with incomplete data were excluded, missing or unrecorded variables may still have influenced the results. Second, this was a single-center study in a predominantly Saudi cohort (86.7%), which may limit generalizability to more diverse populations and healthcare settings. Multicenter studies across varied care models are needed to validate these complications and readmission estimates and improve risk prediction across practice environments. Third, certain potentially relevant factors, including diabetes mellitus subtype, duration of diabetes, severity of peripheral artery disease, neuropathy status, and type of diabetes therapy, were not consistently available and therefore could not be included in the analysis. This limitation was primarily due to the emergent nature of many admissions, in which detailed chronic disease histories were not available at presentation. Fourth, complication ascertainment relied on retrospective EMR data and is therefore subject to documentation and misclassification bias, particularly for clinically diagnosed outcomes such as infection and delirium. To improve reproducibility, we clarified our operational definitions and data sources; however, residual variability in how complications are documented across clinicians and settings cannot be excluded. Fifth, although we addressed anatomic heterogeneity by categorizing amputations as major versus minor and including the amputation category in regression modelling, residual heterogeneity within these categories (e.g., toe vs. below-knee vs. above-knee) may persist and attenuate level-specific associations. Finally, the study was not designed to evaluate secular trends in outcomes over time or to isolate potential COVID-19–era effects; therefore, dedicated follow-on analyses with standardized time-period definitions and appropriate adjustment for time-at-risk would be required to address this question.

## 5. Conclusions

In this decade-long retrospective cohort study of patients undergoing diabetic foot amputations at a tertiary care center, postoperative complications were highly prevalent, affecting more than half of the cohort. Infectious complications were the most common adverse events, and substantial rates of readmission, re-amputation, ICU admission, and mortality were observed, underscoring the profound systemic vulnerability of this population even after definitive surgical intervention. These findings support the need for early risk stratification, targeted perioperative optimization, and structured multidisciplinary follow-up to reduce postoperative morbidity and recurrent healthcare utilization in this high-risk population.

## Figures and Tables

**Table 1:** Demographic and Clinical Characteristics.

Demographics		Value	Percentage/IQR	*p* Value
**Total patient**	*n*	437	-	-
**Age**	Whole population (mean SD)	62.0 (11.8)	-	-
	Male age (mean SD)	61.62 (12.29)	-	0.277
	Female age (mean SD)	62.92 (10.88)	-	
**Gender**	Male	287	65.68%	<0.001
	Female	150	34.32%	
**Nationality**	Saudi	379	86.73%	<0.05
	Non-Saudi	58	13.27%	
**Marital Status**	Married	301	68.88%	<0.001
	Single	128	29.29%	
	Divorced	7	1.60%	
	Widowed	1	0.23%	
**Age groups**	18–39	13	2.97%	<0.001
	40–59	168	38.44%	
	60–79	220	50.34%	
	80 and older	36	8.24%	
**Comorbidities**	Hypertension	340	78.52%	-
	Dyslipidemia	184	43.09%	
	Coronary Artery Disease	183	42.26%	
	Chronic Kidney Disease	123	28.41%	
	Others	225	51.49%	
**Anthropometric measurements**	Weight, Kg	78.00	(65.00–90.00)	-
	Height, cm	165.00	(158.00–170.00)	
	BMI, Kg/m^2^	28.14	(24.41–32.40)	

Data are presented as mean (standard deviation, SD), median (interquartile range, IQR), or *n* (%), as appropriate. BMI, body mass index.

**Table 2:** Laboratory and Amputation-related Details.

Variables	Category	*n*	Percentage/IQR	*p* Value
**Level of Amputation**	Above Knee Amputation	76	17.4%	<0.001
	Below Knee Amputation	139	31.8%	
	Toe Amputation	200	45.8%	
	Metatarsal Amputation	16	3.6%	
	Others	6	1.3%	
**Type of admission**	Emergency	347	79.7%	<0.001
	Elective	88	20.2%	
**Surgical Specialty**	Vascular Surgery	240	54.9%	<0.001
	Orthopedic Surgery	121	27.6%	
	Plastic Surgery	21	4.8%	
	General Surgery	55	12.5%	
**Laboratories**	HbA1c	9.20	(7.70–10.88)	-
	Hemoglobin	103.00	(92.00–116.00)	
	Creatinine	98.00	(70.00–185.50)	
	Albumin	24.75	(19.76–30.21)	
	WBCs	12.70	(8.72–17.80)	
	Lymphocyte	1.70	(1.20–2.40)	
	Neutrophils	9.50	(5.60–14.60)	
	ESR	87.50	(54.25–109.00)	

Interquartile range (IQR), ESR, erythrocyte sedimentation rate; HbA1c, glycated hemoglobin; WBCs, white blood cells. Units: HbA1c (%); Hemoglobin (g/L); Creatinine (µmol/L); Albumin (g/L); WBCs, Lymphocytes, and Neutrophils (×10^9^/L); ESR (mm/h).

**Table 3:** Risk Factors of Postoperative Complications.

Parameters		Any Postoperative Complications (*n*= 228)	No Postoperative Complications (*n* = 209)	*p*-Value
**Age**	Mean (SD)	62.10 (11.63)	62.04 (12.06)	0.959
**Gender**	Male	153 (67.11%)	134 (64.11%)	0.511
	Female	75 (32.89%)	75 (35.89%)	
**BMI**	Median (IQR)	28.24 (24.21–32.39)	28.00 (24.73–32.41)	0.876
**Admission Type**	Elective	37 (16.37%)	51 (24.40%)	0.037
	Emergency	189 (83.63%)	158 (75.60%)	
**Comorbidities**	Hypertension	189 (82.89%)	151 (72.25%)	0.007
	Dyslipidemia	105 (46.05%)	79 (37.80%)	0.106
	CAD	112 (49.12%)	71 (33.97%)	<0.001
	CKD	79 (34.65%)	44 (21.05%)	<0.001
	Others	134 (58.77%)	91 (43.54%)	0.002
**Laboratories**	HbA1c	9.20 (7.70–10.78)	9.15 (7.80–11.03)	0.603
	Hemoglobin	102.00 (90.00–114.00)	105.00 (93.00–122.00)	0.005
	Creatinine	118.50 (79.75–212.25)	85.00 (63.00–124.00)	<0.001
	Albumin	22.90 (18.97–27.96)	27.28 (21.17–32.00)	<0.001
	WBCs	13.60 (9.90–18.95)	11.60 (8.00–15.70)	<0.001
	Lymphocyte	1.60 (1.10–2.30)	1.80 (1.30–2.65)	0.015
	Neutrophils	10.75 (6.78–15.85)	7.84 (4.80–12.05)	<0.001
	ESR	83.00 (51.50–109.50)	90.00 (62.00–107.00)	0.393
**Level of Amputation**	Toe Amputation	95 (41.67%)	105 (50.24%)	0.338
	Above Knee Amputation	43 (18.86%)	33 (15.79%)	
	Below Knee Amputation	79 (34.65%)	60 (28.71%)	
	Metatarsal Amputation	9 (3.95%)	7 (3.35%)	
	Others	2 (0.88%)	4 (1.91%)	
**LOS**	Median (IQR)	26.09 (14.32–47.07)	15.38 (8.38–29.8)	<0.001

Data are presented as mean (SD), median (interquartile range, IQR), or *n* (%), as appropriate. BMI, body mass index; CAD, coronary artery disease; CKD, chronic kidney disease; HbA1c, glycated hemoglobin; WBC, white blood cell count; ESR, erythrocyte sedimentation rate; LOS, length of hospital stay. Units: HbA1c (%); Hemoglobin (g/L); Creatinine (µmol/L); Albumin (g/L); WBCs, Lymphocytes, and Neutrophils (×10^9^/L); ESR (mm/h).

## Data Availability

All data are available on request from the corresponding author.

## Supplementary Materials

The following supporting information can be downloaded at: https://www.mdpi.com/article/10.3390/medicina62030525/s1, Table S1: Operational definitions and ascertainment of postoperative complications; Table S2: Postoperative outcomes by amputation level (minor vs. major); Table S3: Predictors of postoperative complications. CAD, coronary artery disease; CI, confidence interval; CKD, chronic kidney disease; OR, odds ratio; WBCs, white blood cells.

## Abbreviations

The following abbreviations are used in this manuscript:

DM	Diabetes mellitus
DFUs	Diabetic Foot Ulcers
IRB	Institutional Review Board
EMRs	Electronic Medical Records
BMI	Body Mass Index
CAD	Coronary Artery Disease
CKD	Chronic Kidney Disease
HbA1c	Glycated Hemoglobin
WBCs	White Blood Cell Counts
ESR	Erythrocyte Sedimentation Rate

## References

[1] Sun H., Saeedi P., Karuranga S., Pinkepank M., Ogurtsova K., Duncan B.B., Stein C., Basit A., Chan J.C.N., Mbanya J.C. (2022). IDF Diabetes Atlas: Global, regional and country-level diabetes prevalence estimates for 2021 and projections for 2045. Diabetes Res. Clin. Pract..

[2] Francia P., Gualdani E., Policardo L., Bocchi L., Franconi F., Francesconi P., Seghieri G. (2022). Mortality Risk Associated with Diabetic Foot Complications in People with or without History of Diabetic Foot Hospitalizations. J. Clin. Med..

[3] Yazdanpanah L., Shahbazian H., Nazari I., Arti H.R., Ahmadi F., Mohammadianinejad S.E., Cheraghian B., Hesam S. (2018). Incidence and Risk Factors of Diabetic Foot Ulcer: A Population-Based Diabetic Foot Cohort (ADFC Study)-Two-Year Follow-Up Study. Int. J. Endocrinol..

[4] Lepäntalo M., Apelqvist J., Setacci C., Ricco J.B., de Donato G., Becker F., Robert-Ebadi H., Cao P., Eckstein H.H., De Rango P. (2011). Chapter V: Diabetic foot. Eur. J. Vasc. Endovasc. Surg..

[5] Zhang P., Lu J., Jing Y., Tang S., Zhu D., Bi Y. (2017). Global epidemiology of diabetic foot ulceration: A systematic review and meta-analysis. Ann. Med..

[6] Everett E., Mathioudakis N. (2018). Update on management of diabetic foot ulcers. Ann. N. Y. Acad. Sci..

[7] Holman N., Young R.J., Jeffcoate W.J. (2012). Variation in the recorded incidence of amputation of the lower limb in England. Diabetologia.

[8] El-Kebbi I.M., Bidikian N.H., Hneiny L., Nasrallah M.P. (2021). Epidemiology of type 2 diabetes in the Middle East and North Africa: Challenges and call for action. World J. Diabetes.

[9] Al Dawish M.A., Robert A.A., Braham R., Al Hayek A.A., Al Saeed A., Ahmed R.A., Al Sabaan F.S. (2016). Diabetes Mellitus in Saudi Arabia: A Review of the Recent Literature. Curr. Diabetes Rev..

[10] Fan L., Wu X.J. (2021). Sex difference for the risk of amputation in diabetic patients: A systematic review and meta-analysis. PLoS ONE.

[11] Rossaneis M.A., Haddad Mdo C., Mathias T.A., Marcon S.S. (2016). Differences in foot self-care and lifestyle between men and women with diabetes mellitus. Rev. Lat. Am. Enfermagem..

[12] Polikandrioti M., Vasilopoulos G., Dousis E., Gerogianni G., Panoutsopoulos G., Dedes V., Koutelekos I. (2021). Quality of Life and Self-care Activities in Diabetic Ulcer Patients, Grade 3: Gender Differences. J. Caring Sci..

[13] Yao Y., Chen L., Qian Y. (2024). Age Characteristics of Patients With Type 2 Diabetic Foot Ulcers and Predictive Risk Factors for Lower Limb Amputation: A Population-Based Retrospective Study. J. Diabetes Res..

[14] Bischoff C.J., Scheiderer J.A., Garlapaty A.R., Li J., Leary E.V., Schweser K., Stannard J.P., Della Rocca G.J., Kfuri M., Crist B.D. (2025). Outcomes and risk factors for complications, readmissions, and reoperations following below-knee amputation: A comprehensive analysis beyond the 30-day window. Eur. J. Orthop. Surg. Traumatol..

[15] Hamudu H., Nyawale H., Silago V., Mirambo M.M., Chalya P.L., Mshana S.E. (2025). Incidence, bacteriological profile and predictors of surgical site infections following limb amputation at Bugando medical centre and Sekou toure referral regional hospital, Mwanza, Tanzania. J. Orthop. Surg. Res..

[16] Dawi J., Tumanyan K., Tomas K., Misakyan Y., Gargaloyan A., Gonzalez E., Hammi M., Tomas S., Venketaraman V. (2025). Diabetic Foot Ulcers: Pathophysiology, Immune Dysregulation, and Emerging Therapeutic Strategies. Biomedicines.

[17] Kim J. (2023). The pathophysiology of diabetic foot: A narrative review. J. Yeungnam Med. Sci..

[18] Mills J.L., Beckett W.C., Taylor S.M. (1991). The diabetic foot: Consequences of delayed treatment and referral. South. Med. J..

[19] Almohammadi A.A., Alnashri M.M., Abdulrahman THarun R., Alsamiri S.M., Alkhatieb M.T. (2022). Pattern and type of amputation and mortality rate associated with diabetic foot in Jeddah, Saudi Arabia: A retrospective Cohort Study. Ann. Med. Surg..

[20] Shatnawi N.J., Al-Zoubi N.A., Hawamdeh H.M., Khader Y.S., Garaibeh K., Heis H.A. (2018). Predictors of major lower limb amputation in type 2 diabetic patients referred for hospital care with diabetic foot syndrome. Diabetes Metab. Syndr. Obes..

[21] Chahrour M.A., Kharroubi H., Al Tannir A.H., Assi S., Habib J.R., Hoballah J.J. (2021). Hypoalbuminemia is Associated with Mortality in Patients Undergoing Lower Extremity Amputation. Ann. Vasc. Surg..

[22] Wong L.H., Woelber E., Wyland A., Arakawa J., Gundle K.R., Working Z.M., Meeker J.E. (2021). Is Reoperation Higher Than Expected after Below-the-knee Amputation? A Single-center Evaluation of Factors Associated with Reoperation at 1 Year. Clin. Orthop. Relat. Res..

[23] Brix A.T.H., Rubin K.H., Nymark T., Schmal H., Lindberg-Larsen M. (2024). Length of hospital stay and readmissions after major lower extremity amputation: A Danish nationwide registry study. Acta Orthop..

[24] Kayssi A., de Mestral C., Forbes T.L., Roche-Nagle G. (2016). Predictors of hospital readmissions after lower extremity amputations in Canada. J. Vasc. Surg..

[25] Hennessy C., Abram S., Loizou C., Brown R., Sharp B., Kendal A. (2025). Mortality, Re-Amputation, and Postoperative Complication Rates Following 28,000 Below-Knee Amputations in Diabetic Patients in England: A National Population Study, 2002–2022. Orthop. Proc..

[26] Kumbhar S., Bhatia M. (2024). Advancements and best practices in diabetic foot Care: A comprehensive review of global progress. Diabetes Res. Clin. Pract..

[27] Tavakoli M., Petropoulos I.N., Malik R.A. (2013). Corneal confocal microscopy to assess diabetic neuropathy: An eye on the foot. J. Diabetes Sci. Technol..

[28] Quattrini C., Tavakoli M., Jeziorska M., Kallinikos P., Tesfaye S., Finnigan J., Marshall A., Boulton A.J., Efron N., Malik R.A. (2007). Surrogate markers of small fiber damage in human diabetic neuropathy. Diabetes.

[29] Selvarajah D., Kar D., Khunti K., Davies M.J., Scott A.R., Walker J., Tesfaye S. (2019). Diabetic peripheral neuropathy: Advances in diagnosis and strategies for screening and early intervention. Lancet Diabetes Endocrinol..

[30] Pušnik L., Gabor A., Radochová B., Janáček J., Saudek F., Alibegović A., Serša I., Cvetko E., Umek N., Snoj Ž. (2025). High-Field Diffusion Tensor Imaging of Median, Tibial, and Sural Nerves in Type 2 Diabetes With Morphometric Analysis. J. Neuroimaging.

