# Supplementary material for: Outcomes and Laboratory Predictors of Complications Following Diabetic Foot Amputations: A Ten-Year Tertiary Care-Center Experience
Source: Medicina (Kaunas). 2026 Mar 12;62(3):525. doi: 10.3390/medicina62030525 (PMC13027830; doi:10.3390/medicina62030525)
Supplement: Supplementary file 1 [file medicina-62-00525-s001.zip › medicina-4104015-supplementary.pdf]

Table S1: Operational definitions and ascertainment of postoperative complications

Complication domain	Specific event(s)	Operational definition used in this study	Data source(s)
Infectious	SSI	(e.g., clinician-documented SSI ± culture/imaging/antibiotics)	Notes / micro / antibiotics
	Osteomyelitis	(e.g., clinician-documented ± imaging/culture)	Notes / radiology / micro
Renal	AKI	ΔCr ≥26.5 μmol/L/48h or ≥1.5× baseline/7d	Notes / labs
CV	MI	(e.g., clinician diagnosis ± ECG/troponin)	Notes / labs
Neuro	Stroke	(e.g., clinician diagnosis ± CT/MRI)	Notes / imaging
VTE	DVT/PE	(e.g., imaging-confirmed)	Imaging
Neuropsychiatric	Delirium	(e.g., clinician-documented delirium/acute confusional state)	Notes

AKI, acute kidney injury; Cr, creatinine; CT, computed tomography; CV, cardiovascular; DVT, deep vein thrombosis; ECG, electrocardiogram; MI, myocardial infarction; MRI, magnetic resonance imaging; PE, pulmonary embolism; SSI, surgical site infection; VTE, venous thromboembolism.

Table S2: Postoperative outcomes by amputation level (minor vs major)

Outcomes	Minor (n= 222)	Major (n= 215)	*P*-value
Postoperative complications	106 (47.75%)	122 (56.74%)	0.060
Readmission	122 (54.95%)	99 (46.05%)	0.063
ICU Admission	39 (17.57%)	85 (39.53%)	<0.001
Re-amputation	43 (19.37%)	58 (26.98%)	0.136
Mortality	25 (11.26%)	52 (24.19%)	0.002

Table S3: Predictors of postoperative complications

Parameters		OR (95% CI)	*P*-value	Adjusted OR (95% CI)	Adjusted *P*-value
Age [Year]		1.000 (0.985 – 1.016)	0.959	-	-
Gender	Male	Reference			
	Female	0.876 (0.590 – 1.300)	0.511	-	-
Admission Type	Elective	Reference		Reference	
	Emergency	1.649 (1.027 – 2.646)	0.038	1.260 (0.748 – 2.120)	0.385
Comorbidities (No as a reference)	Hypertension	1.733 (1.090 – 2.757)	0.020	1.284 (0.761 – 2.165)	0.349
	CAD	1.822 (1.237 – 2.685)	0.002	1.439 (0.930 – 2.227)	0.102
	CKD	1.940 (1.261 – 2.985)	0.003	1.335 (0.735 – 2.428)	0.343
	Others	1.849 (1.264 – 2.703)	0.002	1.497 (0.993 – 2.256)	0.054
Laboratories	Hemoglobin	0.983 (0.973 – 0.993)	0.001	0.997 (0.985 – 1.009)	0.608
	Creatinine	1.002 (1.001 – 1.003)	0.003	1.001 (0.999 – 1.002)	0.370
	Albumin	0.927 (0.901 – 0.955)	<0.001	0.944 (0.913 – 0.976)	<0.001
	WBCs	1.050 (1.023 – 1.078)	<0.001	1.006 (0.964 – 1.049)	0.792
	Lymphocyte	0.933 (0.826 – 1.053)	0.260	-	-
	Neutrophils	1.063 (1.032 – 1.094)	<0.001	1.030 (0.980 – 1.081)	0.243
Amputation	Major	1.436 (0.985 – 2.093)	0.060	1.197 (0.769 – 1.863)	0.425
	Minor	Reference		Reference	

CAD, coronary artery disease; CI, confidence interval; CKD, chronic kidney disease; OR, odds ratio; WBCs, white blood cells.
